# 

**Published:** 1902-07-12

**Authors:** 


### NOTICE TO CORRESPONDENTS.

All MSS., Letters, Books for Review, and other matters intended for the Editor should be addressed The Editor, "The Hospital," The Hospital Building, 28 & 29 Southampton Street, Strand, W.C.

The Editor cannot undertake to return rejected MS., even when accompanied by stamped directed envelope.

### Notice to Advertisers and Subscribers.

All Advertisements, Orders for Copies of The Hospital, and Business Communications should be addressed to **THE MANAGER, (Not to the Editor)**, The Hospital, 28 & 29 Southampton Street, Strand, London, W.C.

The Cost of Subscription, post free, is as follows.—Per week, 2½d.; per quarter, 2s. 8d.; per half-year, 5s. 3d.; per annum, 10s. 6d. Foreign:—Per annum, 15s. 2d., payable, in all cases, in advance.

**NOTICE.—Vol. XXXI. of "The Hospital" (October 1901, to March 1902), in handsome cloth binding is now on sale at the office of this Journal, price, 7s. 6d. Cases for binding the Numbers contained in this Volume 1s. 6d. Index to Vol. XXXI., 2½d., post free.**

**The Monthly Part for July is now ready. Price 6d., by post 8d.**

### BOOKS RECEIVED.

Geo. Gill and Sons.

"Hygiene: a Manual of Personal and Public Health." By Arthur Newsholme, M.D., F.R.C.P.

Partridge and Cooper.

Hymn composed for the Coronation. Words by A. W. Letts. Music by the Rev. A. Wellesley Batson, Mus. Bac.

Rebman, Limited.

"Christian Science, Medicine and Occultism." By Albert Moll, M.D.Berlin. Translated by F. J. Rebman.

T. B. Browne.

"In Peaceful Africa."

Scientific Press.

"The Human Body: its Personal Hygiene and Practical Physiology."

Baillière, Tindall and Cox.

"A Manual of Practical Medical Electricity, the Roentgen Rays and Finsen Light." By Dawson Turner, B.A., M.D., F.R.C.P.Edin., M.R.C.P.Lond.

### SCRAPS AND GLEANINGS.

THE new isolation hospital at Oakley Hill, Bedford, which was recently opened for the reception of patients, has cost £10,000 to put up, including cost of site. It consists of two pavilions containing altogether 18 beds.

DURING the quarter ending March 31st, 1902, the district nurse attached to the Bury Cottage Hospital paid 550 visits. The honorary secretary reported at the meeting of the hospital management committee, held recently, that the funds were very low, and the District Council had been approached with a view to their handing over to the hospital any surplus there might be from the Coronation celebration funds.

DURING last year 962 in-patients were treated at St. Bartholomew's Hospital, Rochester, notwithstanding the fact that during the early part of 1901 some of the beds were not available owing to the sanitary alterations being made in the hospital. There were 13,637 out-patients who made 42,408 attendances. Further alterations and improvements in the building have now been decided upon, and the work has been put in hand to provide a new and modern operating theatre, a lift for patients, steam power for the laundry. Plans for providing more beds for patients, a children's ward, and proper accommodation for the nursing staff are being prepared, and the work is to be pushed on without delay.

### THE STUDENT OF MEDICINE.

### APPOINTMENTS.

**Gilbert James Arnold**, F.R.C.S.Eng., L.R.C.P.Lond., appointed Honorary Surgeon to the Torbay Hospital, Torquay. **J. Blamey**, M.R.C.S.Eng., L.S.A., re-elected Medical Officer for Perranwell. **Alexander Whyte Cassie**, M.A., M.B., Ch.B.Aberd., appointed Medical Officer of Forres Leanchoil Hospital. **James Collier**, M.D., M.R.C.P.Lond., appointed Assistant Physician to the National Hospital, Queen Square, W.C. **J. Corlis**, M.D.Cantab., appointed Public Vaccinator for the Urban and Suburban Districts of Menzies, Western Australia. **Henry Moyer Cyril Dalton**, M.B., C.M.Glasg., appointed Government Medical Officer and Vaccinator at Murrumburrah, New South Wales, Australia. **G. C. Elliott**, M.B., C.M.Edin., appointed House Surgeon to the Staffordshire General Infirmary. **C. O. Hawthorne**, M.D.Glasg., M.R.C.P.Lond., appointed Lecturer on Forensic Medicine in the London School of Medicine for Women. **H. M. Hewlett**, L.R.C.P., M.R.C.S.Edin., L.F.P.S.Glasg., appointed Honorary Assistant Pathologist to the Benevolent Asylum, Melbourne, Victoria, Australia. **Alfred William Hill**, M.D.Brux., appointed Honorary Surgeon to the Ear and Throat Department at the Adelaide Hospital. South Australia. **Thomas Horton**, M.D., B.S.Durham, L.R.C.P.Lond., M.R.C.S., appointed Honorary Surgeon to the Torbay Hospital, Torquay. **Theo. J. Ick**, M.B.Melb., appointed District Medical Officer and Public Vaccinator at Jarrahdale, Western Australia. **Iain Jefferiss**, M.R.C.S.Eng., L.R.C.P.Lond., appointed First Assistant Medical Officer to the Hendon Sick Asylum. **John King**, M.R.C.S., L.R.C.P.Edin., J.P., reappointed Medical Officer of Health for Stratton and Bude (Cornwall). **Thos. D. Lister**, M.D., M.R.C.P.Lond., appointed Assistant Physician to the Mount Vernon Hospital for Consumption and Diseases of the Chest, Hampstead and Northwood. **Charles Kinnaird Mackellar**, M.B., C.M.Glasg., appointed President of the State Children Relief Board, New South Wales, Australia. **Herbert C. Male**, M.D.Edin., appointed an Honorary Medical Officer to the Croydon General Hospital. **L. J. Miskin**, M.B.Lond., M.R.C.S.Eng., appointed Acting District Medical Officer and Public Vaccinator, Coolgardie, Western Australia. **M. J. O'Brien**, L.R.C.P., L.R.C.S.I., appointed Certifying Surgeon under the Factory Act for the Mountmellick District of Queen's County. **Mary E. Phillips**, M.B.Lond., appointed Second Assistant Medical Officer at the London Sick Asylum at Hendon. **Allan E. Randell**, M.B., B.S.Melb., appointed Officer of Health at Melville, Western Australia. **Matthew Alexander Reid**, L.R.C.P.Lond., appointed Public Vaccinator for the Metropolitan District of Melbourne, Australia. **Thomas Thyne**, M.D.Edin., appointed Medical Officer for the Mill Corner and Cockfosters District of Enfield Parish. **Hugh P. Wright**, M.B., Ch.B.Glasg., appointed House Surgeon to Teignmouth Hospital, South Devon. **Dr. H. B. Wyman**, appointed Assistant Medical Officer at Cheadle.

**"The Hospital" Institutional Library.**

"Hospitals and Asylums of the World." 4 Vols., with a Portfolio of Plans, complete £12 12s.

"Burdett's Hospitals and Charities, 1902." 5s.

"Cottage Hospitals, General, Fever, and Convalescent." 10s. 6d.

"Hospital Expenditure: The Commissariat." 2s. 6d.

"A Legal Handbook for the Use of Hospital Authorities.' 2s. 6d.

"The Cottage Hospital Case Book or Register of Patients." 10s. and 12s. 6d.

"The Furnishing and Appliances of a Cottage Hospital." 6d.

All these are published by the SCIENTIFIC PRESS, Ltd., and may be obtained through any bookseller, or direct from the publishers, 28 and 29 Southampton Street, Strand, London, W.C.

